# Expanding Training Data for Structure-Based Receptor–Ligand Binding Affinity Regression through Imputation of Missing Labels

**DOI:** 10.1021/acsomega.3c05931

**Published:** 2023-10-26

**Authors:** Paul G. Francoeur, David R. Koes

**Affiliations:** Department of Computational and Systems Biology, University of Pittsburgh, Pittsburgh, Pennsylvania 15260, United States

## Abstract

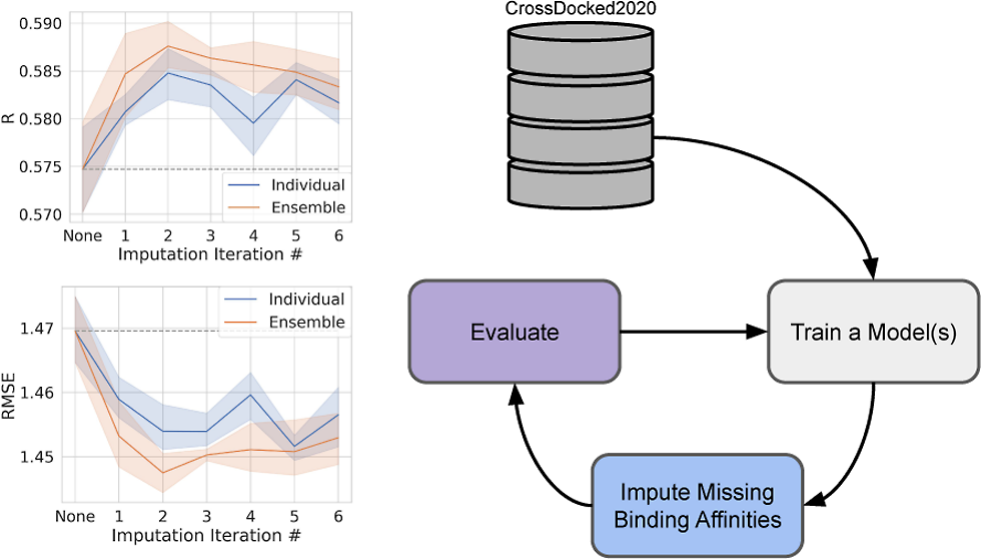

The success of machine learning is, in part, due to a large volume of data available to train models. However, the amount of training data for structure-based molecular property prediction remains limited. The previously described CrossDocked2020 data set expanded the available training data for binding pose classification in a molecular docking setting but did not address expanding the amount of receptor–ligand binding affinity data. We present experiments demonstrating that imputing binding affinity labels for complexes without experimentally determined binding affinities is a viable approach to expanding training data for structure-based models of receptor–ligand binding affinity. In particular, we demonstrate that utilizing imputed labels from a convolutional neural network trained only on the affinity data present in CrossDocked2020 results in a small improvement in the binding affinity regression performance, despite the additional sources of noise that such imputed labels add to the training data. The code, data splits, and imputation labels utilized in this paper are freely available at https://github.com/francoep/ImputationPaper.

## Introduction

The success of deep learning is due in part to training large models on large data sets. A recent example of this phenomenon in the protein structure prediction task is the 93 million parameter model AlphaFold, which was trained on the 204,104 structures available in the protein data bank and 355,993 unlabeled sequences from Uniclust30.^1^ For structure-based predictions for receptor–ligand binding affinity, there is much less data available. A common data set for this task is the PDBbind,^2^ containing 23,496 entries in the latest 2020 version.

It is desirable to expand the available training data for structure-based models, which, in turn, can enable the use of more expressive models with higher parameter counts for this task. Additionally, since it is both time-consuming and expensive to generate both a crystal structure and binding affinity measurement for a given receptor–ligand pair, it makes sense to explore in silico methods to expand the training data. In prior work, we introduced the CrossDocked2020 data set, which expands the available pose data for binding complexes through cross-docking.^3^ We made the assumption that ligands will bind to similar receptors in order to group the PDB into binding pocket classes and combinatorially expand the receptor–ligand complexes by docking each ligand in a pocket class to each receptor in the said pocket class. This increased the number of docked binding poses from ∼200,000 generated when docking the PDBbind general set to ∼22.5 million poses in CrossDocked2020.^3^ This expanded data set was used to train the convolutional neural network (CNN) models for protein–ligand scoring^4^ that are used by GNINA docking software.^5^

While CrossDocked2020 massively expands the amount of pose data available for training, a shortcoming of this approach is that it does not expand the amount of binding affinity data. The binding affinity between a protein and a ligand is a measure of how strongly the two molecules interact and is a common modeling task. There have been several different approaches to utilizing ML and other modeling techniques for binding affinity regression.^3,6−17^ CrossDocked2020 sources its binding affinity labels from the PDBbind general set, but all of its possible receptor–ligand pairs from Pocketome.^18^ This results in the existence of ligands in CrossDocked2020 that do not have a binding affinity label as they are not included in the PDBbind general set. The group of unlabeled ligands is the majority of CrossDocked2020 since only ∼40% of CrossDocked2020 has a binding affinity label. For binding affinity predictions, models trained on CrossDocked2020 observe the same distribution of binding affinity training data as models trained on the PDBbind general set. In this work, we investigate the effect of various imputation strategies for the missing binding affinity labels on the model performance.

Imputation is the process of replacing missing data with estimated values.^19,20^ This is especially attractive for ML models as, if the imputed labels are readily available, then there is a negligible impact on training time and compute resources to train the final model. There are several approaches to imputing missing data. The simplest is to delete or ignore data points with missing features (i.e., not do imputation). This is the approach previously taken when training models on the CrossDocked2020 data.^3^ The binding affinity loss is applied only to complexes that have a labeled binding affinity. This approach can introduce extra bias into models, especially if the missing data are not randomly distributed.^21^ The next easiest method of imputation is known as “simple imputation.” It entails replacing the missing values by a single quantifiable attribute of the nonmissing values (e.g., mean, median, and mode). Unfortunately, these methods may produce extra bias or unrealistic results on high-dimensional data sets.^22^ We cannot utilize “simple imputation,” since we know that our labels are a function of our high-dimensional input data (atom positions in 3D space).

Thus, we turn to the regression imputation. In this form of imputation, a model is fit to the known data points and then used to assign the imputed labels to the missing data. There are several approaches to this type of imputation, from the statistical, such as a weighted quantile regression, to more machine learning (ML)-inspired approaches like k-nearest neighbors (k-NN), support vector machines, random forests, and so forth.^20,21^ ML-based imputation approaches have been successful across the medical field, showing promise in imputing Medical Expenditure Panel Surveys,^23^ or being utilized in clinical decision making.^24^ To the best of our knowledge, an ML approach to imputing missing receptor–ligand binding affinities has not been studied. The closest is a study by Rubinsteyn et al.,^25^ who examined a variety of methods for imputing binding affinities for peptide-major histocompatibility complex (MHC) interactions. These methods were not ML-based and predicted a singular class of binding affinity interactions between the MHC class of receptors and their peptide substrates,^25^ and so are not a general model for protein–ligand binding affinity prediction.

The most common ML models employed in imputation are k-NN models and random forest models.^20^ Nearest neighbor algorithms require a meaningful similarity metric. Garcia-Hernandez et al.^26^ show that fingerprint-based similarity metrics can incorrectly measure the similarity between molecules and suggest a similarity based on graph edit distance. However, for our large data set, there is considerable computational expense in computing these similarity metrics. Additionally, we require more than a ligand similarity metric since the receptors and the interactions it makes with the ligand are also important to our binding pose and affinity predictions. It is unclear what similarity metric could capture each of these unique features. We also know that our CNN-based models perform similarly to other random forest-based methods on predicting receptor–ligand binding affinity.^3^ In this work, we investigate using our CNN model architecture and an ensemble of these CNN models to impute the missing binding affinities.

## Methods

Here, we describe our 3D grid-based CNN model architecture, the data set we are utilizing for our experiments, and the training procedure utilized for each experiment.

### Model Architecture

We utilized the Def2018 model previously described.^3^ Briefly, it is a 3D CNN consisting of a 2 × 2 × 2 average pool, followed by a 3 × 3 × 3 convolution and ReLU, followed by a 1 × 1 × 1 convolution and ReLU layer. This pool-convolution-convolution block is repeated once and then followed by a final 2 × 2 × 2 average pool and 3 × 3 × 3 convolution plus ReLU layer. The convolution process results in a total of 6 × 6 × 6 × 128 features, which are passed to two output fully connected layers. These output layers predict the binding affinity and pose score. The input to our model is a 24 × 24 × 24 Å grid at 0.5 Å resolution of a continuous Gaussian density of 14 ligand and 14 receptor atom types. These grids are generated on the fly by libmolgrid.^27^

### Data Set

We utilize the CrossDocked2020v1.3 data set, following the same clustered cross-validation splits utilized in the CrossDocked2020 publication.^3^ Our previous work utilized CrossDocked2020v1.0.^3^ The version upgrades fixed several receptor structures that were flattened in the original data set (version 1.1), and fixed several ligands that had the double bonds removed from their aromatic rings (version 1.2). Version 1.3 fixed an issue where multiple ligands on different chains would be downloaded into the same file, fixed several misaligned receptors and ligand structures, and reduced the Pocketome definition of similar receptors to the nonredundant set in order to remove entries in pockets with a large number of receptors present that did not provide meaningful information when training. CrossDocked2020v1.3 contains 2900 binding pockets, consisting of 17,815 pocket–ligand pairs, and a total of 22,566,449 poses. Of these poses, 13,301,254 (58.9%) have unlabeled binding affinity data. The data set was generated by docking ligands for a given receptor into other receptors in the same pocket, as defined by Pocketome.^18^ Pocketome groups the Protein Data Bank into groups, called pockets, through a similarity of binding sites of the receptors. This means that even though there are multiple receptor–ligand pairs in a given pocket, each pocket can be treated as a single target. The additional noise through cross-docking in this case is due to docking ligands into a binding pocket that is only slightly different from its cognate crystal orientation instead of docking the ligand into an entirely new target. We label poses as good if their root-mean-square deviation (rmsd) of the docked pose to the crystal pose is less than 2 Å or bad otherwise. If a given ligand has binding affinity data in PDBbind version 2017, we assume that it would have the same binding affinity label for all members of a pocket. These data were clustered by pocket similarity using the ProBiS^28^ algorithm with the *z*-score parameter set to 3.5. Each cluster was then randomly assigned to one of three folds for clustered cross-validation.

In order to perform a secondary test of our imputation procedure on model training, we also utilized the data set published by Tosstorff et al.^29^ This data set, referred to as the “Roche” data set, contains a total of 1162 PDE10A inhibitors with experimentally determined binding affinities. For each of the 1162 molecules, a receptor and docked pose of the molecule is provided in the Supporting Information by Tosstorff et al.^29^ We removed any waters present in the receptor structures. Next, we docked the provided ligand file into its corresponding receptor structure with Gnina using the default settings and requesting 20 poses. We then scored the provided pose of the molecule and each of the docked poses with our model to obtain our predictions.

### Model Training Procedure

Consistent with our prior work,^3^ models were trained using our custom fork of the Caffe deep learning framework^30^ with libmolgrid integration^27^ using the train.py script available at https://github.com/gnina/scripts. Training examples were randomly shuffled, and a batch size of 50 was used. Batches were balanced with respect to class labels (low rmsd vs high rmsd poses), and examples were stratified with respect to the receptor so that targets are sampled uniformly during training. This means that during training, the model will “see” an equivalent number of low rmsd poses and high rmsd poses for each binding pocket, and each binding pocket is sampled evenly. This is achieved by upsampling the data for whatever is lower (e.g., the number of low rmsd poses are upsampled so that during training the model is trained on an equivalent number of low and high rmsd poses, even though high rmsd poses make up 96.3% of the data set). Structures were randomly rotated and translated (up to 6 Å). We utilized the stochastic gradient descent optimizer with an initial learning rate of 0.01 and momentum of 0.9 with a weight decay of 0.001.

We utilized early stopping to terminate training. We monitor the performance on a reduced version of the training set, containing about 200,000 complexes. This was generated automatically by the train.py script, with the *percent_reduced* parameter set to 0.132. Every 1000 training iterations, we evaluate the model’s performance on the reduced set. If the performance ceases to improve over 200 evaluations (*step_when*), we lower the learning rate by a factor of 10. The learning rate is allowed to decrease three times (*step_end_cnt*), after which training ends.

Lastly, we implement two loss functions. Pose selection is trained with a logistic loss to distinguish between low rmsd (<2 Å) and high rmsd poses (>2 Å). Affinity prediction is trained with a L2-like pseudo-Huber loss that is hinged when evaluating high rmsd poses. This means the affinity prediction loss is 0 when the model under-predicts the binding affinity on a high rmsd pose, and the loss is penalized for getting both a too high and too low affinity for a low rmsd pose. We note that our model’s loss functions do not take into account whether or not a binding affinity label is imputed. That is, even with an imputed binding affinity label, the model is penalized for predicting a higher binding affinity than the imputed label for a high rmsd pose.

### Experimental Setup

**Figure 1:**
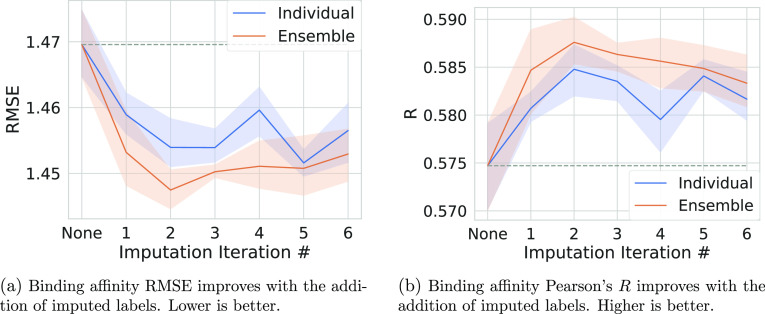
Adding imputed binding affinity labels to the training set provides a small improvement in all predictive tasks. We show the results of six iterations of data imputation and model retraining on affinity labels from CrossDocked2020v1.3. At each data point, we plot the mean of five models trained with different random seeds. The colored area is the 95% confidence interval around the mean calculated via bootstrapping in seaborn. The blue line shows the results of five different random seeds (individual, Table 1). The orange line shows an ensemble approach, taking the mean of the five models as the imputed label of every pose (individual ensemble, Table 1). A dashed gray line is extended from the mean result of training without imputed labels for easier comparison with the baseline.

For each of the following experiments, we train five models, each utilizing a different random seed, on the three-fold clustered cross-validation splits previously described. The general model training schema for each experiment is (1) train and evaluate an initial model ignoring any missing labels, (2) use the trained model and an imputation scheme to impute the missing labels, and (3) train and evaluate a new model on the imputed data. For the individual and individual ensemble, we repeated steps 2–3 until the performance on the test set stopped improving (Figure 1). We then compared models trained with imputation types that had pocket–ligand groupings one a single round of imputation, and for the best-performing model, we repeated steps 2–3 one time.

We investigated several imputation schemes in order to determine their effect on the model binding affinity prediction performance. First, we utilized the simplest imputation type: treating each binding pose as a different example and simply utilizing the predictions of a trained model independently (individual row in Table 1). That is, there was no ensemble between the different seeds in the imputation for the missing data. This results in each seeded model having a different training set from that of one another.

**Table 1:** How a Given Experimental Approach Imputes a Given Pocket–Ligand Complex^a^

imputation type	pocket–ligand grouping	only low rmsd poses
individual	no	no
individual ensemble	no	no
median ensemble	yes	no
median good only ensemble	yes	yes
max ensemble	yes	no
max good only ensemble	yes	yes
min ensemble	yes	no
min good only ensemble	yes	yes

a The imputation types marked with “Individual” create an imputed binding affinity label for every pose. The other imputation types select a single imputed label for every pose for a given pocket–ligand complex. The label selection is carried out by taking the median, maximum, or minimum of the imputed labels for either all poses or only the low rmsd poses of the pocket–ligand complex.

The second imputation type utilized the mean of each of the five models to produce the imputation label for each pose (individual ensemble row in Table 1). This allows for the same training set to be utilized for each seed during training at each round of imputation and produces a distinct binding affinity label for each binding pose in a given pocket–ligand complex. Notably, only the imputed binding affinities have a pose-specific label for the binding affinity, unlike the rest of the training data. It is unclear whether this is desirable or not, so we also investigated a third imputation type.

In the remaining imputation types, we stored the predicted label of every pose for each pocket–ligand complex (rows with pocket–ligand grouping in Table 1). We then took as our imputation label for a single model either the maximum, median, or minimum of these predicted values for every pose in the said pocket–ligand complex. The resulting labels from the five models were then averaged to provide the final imputation label for a given pocket–ligand complex. In contrast to the second imputation scheme, this approach results in a single value being utilized for every pose of a ligand, just as with the experimental data.

A potential flaw with the previously described imputation approach is that we also include the predicted binding affinity for poses that we know are not in the correct binding orientation when calculating the aggregate statistics. For example, there are many more high rmsd poses than low rmsd poses for every pocket–ligand pair. Additionally, we know that, due to the hinge loss when training, our model tends to predict lower binding affinities for high rmsd poses.^3^ Thus, when calculating the minimum and median aggregate statistics for imputation, we artificially lower the calculated number through the inclusion of many poses that are of low quality. In order to alleviate this problem, we also investigate modifying our approach to include only predictions from the low rmsd poses (less than 2 Å from the crystal pose).

Lastly, we characterize how much imputed data are necessary to achieve the maximal performance gain for our best-performing imputation type. We evaluate randomly adding increments of 20% of the imputed binding labels to the training data of the model, that is, training with no imputed labels, 20, 40, 60, 80, or 100% of the imputed labels. This allows us to characterize the effect of adding various levels of imputed data to the training of our models and determine the optimal amount of imputed labels to add to the training data.

To compare these experiments we adopt the same testing criteria and methods described in the initial CrossDocked2020 paper.^3^ Namely, for each pocket–ligand pair, we select a singular pose by selecting the pose with the highest predicted pose score to be our candidate. We then calculate the Pearson’s *R*, the root-mean-squared error (RMSE), and coefficient of determination between the candidate poses binding affinity labels and the predictions from our model; we only evaluate the molecules in the test set that have a binding affinity label. We additionally evaluate the area under the receiver operating characteristic curve (AUC), and the fraction of times that the top-ranked binding pose is less than 2 Å rmsd to the native crystal pose (Top1) to serve as measures of our model’s ability to use the imputed binding affinity data to improve its performance at the orthogonal task of distinguishing bad and good poses.

## Results

In this section, we demonstrate that an imputation approach utilizing our CNN model to predict receptor–ligand binding affinities and using those predictions as training data improves the model performance at both binding affinity regression and binding pose classification. We also show that utilizing an ensemble of predictions for imputation is better than utilizing a single model. Additionally, we demonstrate that utilizing a single imputed value from an ensemble of predictions only from good poses for every pocket–ligand pair further improves the performance at binding affinity prediction. Lastly, we provide evidence that utilizing a roughly equal balance of imputed training data and real training data achieves the maximal model performance.

### Imputation Improves the Model Performance

In our first experiment, we sought to determine whether imputing missing labels improves our CNN’s ability to predict receptor–ligand binding affinity. For this experiment, we utilized the individual and individual ensemble imputation types. Figure 1 shows that utilizing imputed labels indeed improves the model’s performance on binding affinity prediction, with two iterations of training + input maximizing performance gains. Additionally, even though our binding pose classification data remain unchanged, we note that providing the model with imputed binding affinity labels also results in a small improvement on the binding pose classification task (Figure S1).

In our prior work, we demonstrated that our binding affinity predictions were pose dependent, in part due to the Def2018 architecture sharing weights for the binding pose classification and binding affinity regression task.^3^ For this experiment, each pose is also getting an independent binding affinity imputation (e.g., if a particular receptor–ligand complex has 20 poses, there will be 20 imputed affinities). During training, the loss is a combination of affinity loss and classification loss. The extra imputed binding affinity labels can supply more data to the overall loss function as now each pose has both the affinity loss and the classification loss.

However, it is unclear whether utilizing different imputations for every pose is the best approach for performance on the binding affinity regression task. When training with experimental binding affinities, each pose is labeled with the same binding affinity label. This is not true of the imputed labels in this experiment as we impute a different binding affinity label for each docked pose of the complex. This introduces an additional source of information in the training data as the complexes with an imputed binding affinity label have additional noise compared to the known labels. If we trust our model’s ability to predict binding affinity accurately, then it does not make sense to hamper our model with extra noise when training on the imputed labels. On the other hand, this extra source of information could be beneficial to the final trained model’s performance.

### Restricting Imputation to Good Poses Further Improves the Model Performance

**Figure 2:**
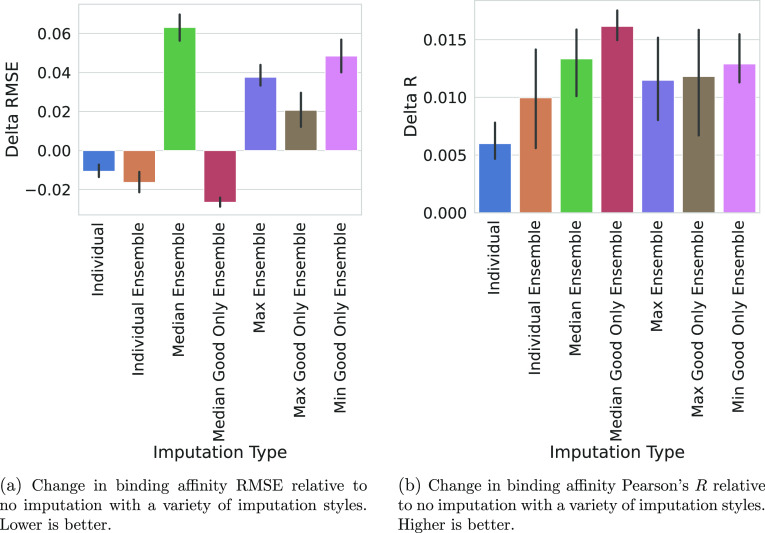
Comparing different binding affinity imputation styles. The performance metrics for five models with different seeds trained with each imputation style were subtracted from the mean performance of training without imputation. The shaded area is the 95% confidence interval of this described value calculated via bootstrapping in seaborn. For each plot, a bar corresponds to a singular imputation style. The first two styles, Individual and Individual_Ensemble, are the same that are used in Figure 1. For the rest of the styles, we select one number for each pocket–ligand pair, either by the median (Med), maximum (Max), or minimum (Min). We then either utilized the Med/Max/Min of all poses, or only the poses labeled good. The Min ensemble results were omitted, due to performing so poorly that they rescaled the plots (delta RMSE 1.674, delta *R* −0.157). The student’s *t*-test for each of these values is reported in Table 2. A dashed gray line is extended from the mean result of training without imputed labels for easier comparisons.

In order to address this question, we investigated utilizing different approaches for generating the imputed labels. To make the data appear similar to the original training data, we used the same imputed binding affinity label for each pocket–ligand pair. We consider three approaches for aggregating the imputed binding affinity labels for a given pocket–ligand pair: the median, maximum, or minimum. We also investigated the effect of this calculation using all possible poses for the pocket–ligand pair or only utilizing the predictions from the low rmsd good poses. We then utilized the ensemble mean of five differently seeded models as our imputed binding affinity label for a given receptor–ligand complex and trained five new models with different seeds on the new data set. The results for a single iteration of imputation and evaluation are shown in Figure 2 and S2.

**Figure 3:**
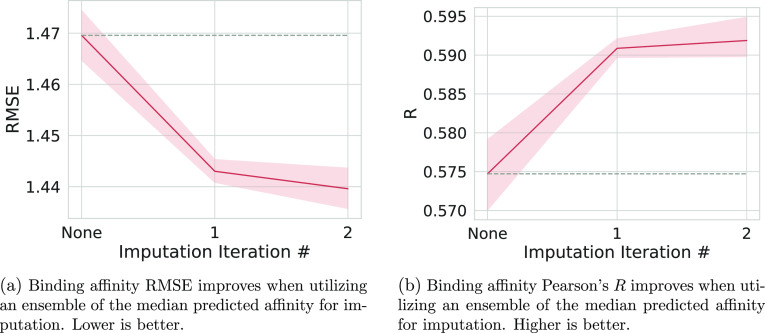
Performance metrics of our best imputation approach, taking the ensemble mean of the median predicted binding affinity for each pocket–ligand complex good pose, for binding affinity regression. The 0th point on the line is the model results after training on the original data set. We then used that model to generate the imputed labels and utilized them to train the model for the first data point. The said model was then used to generate the imputed labels for the second data point’s model’s training. For each point, five models with different seeds were trained from scratch. The shaded area is the 95% confidence interval of the mean calculated via bootstrapping in seaborn. A dashed gray line is extended from the mean result of training without imputed labels for easier comparisons.

All imputation types improved the model’s performance on binding affinity Pearson’s *R*, but only the median good only ensemble improved the predicted binding affinity RMSE. This imputation type also had the best performance gain on the binding affinity regression task compared with training without imputation. As such, we performed another iteration of imputation training, similar to the setup from the prior experiment. The results of this extra round of imputation are shown in Figure 3 and S3. Again, we observe additional performance gain on the second round of imputation, and the gain is minimal compared to the initial gain. Due to this, and the results of Figure 1, we performed no additional rounds of imputation.

We note that while the median good only ensemble provided the best results for the binding affinity prediction task, it performed relatively poorly on the binding pose classification task (Figure S2). The individual ensemble performed the best at having the top-ranked pose have low rmsd (Top1) and was the only approach to achieve a statistically significant improvement between the models trained with imputation and those without (Table S1). Both the individual and individual ensemble imputation types resulted in the best performance gain for AUC (Figure S2).

**Table 2:** Student’s *T*-test *p*-values for the Difference between 0 and 1 Round of Imputation for Each of the Methods^a^

imputation type	RMSE	*R*
individual	0.0144	0.0595
individual ensemble	0.00482	0.0253
ensemble median	1.07 × 10^–6^	0.00234
ensemble good only median	3.43 × 10^–5^	0.000304
ensemble max	2.29 × 10^–5^	0.00863
ensemble good only max	0.00712	0.0144
ensemble min	3.14 × 10^–13^	1.54× 10^–10^
ensemble good only min	4.27 × 10^–5^	0.00202

a This table corresponds to the data utilized to generate Figure 2. Notably, for the binding affinity RMSE, every method results in a statistically significant difference as compared to the case of not performing the imputation. This is not true for all the other metrics, though generally imputation results in a statistically significant difference for binding affinity Pearson’s *R* and AUC while generally failing to produce a statistically significant difference for Top1.

### Balancing Imputed and Known Labels Maximizes Model Learning

**Figure 4:**
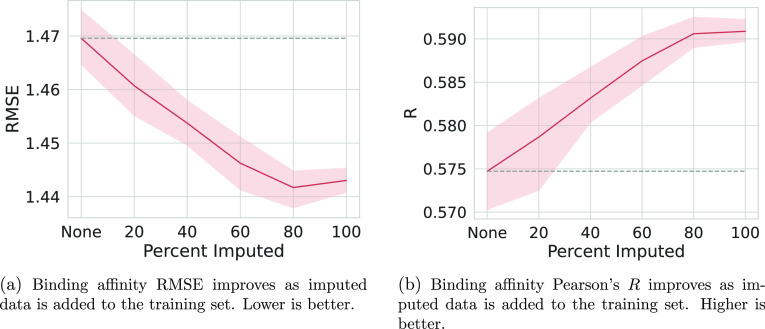
Effect on metrics as a function of successively adding more imputed binding affinities to the training set. Each plot shows the results of five models with different seeds, being trained on successively more of the imputed binding affinity labels. The shaded area is the 95% confidence interval of the mean calculated via bootstrapping in seaborn. Shown are no imputed labels to all of the imputed labels, in increments of 20%. The imputation generation procedure is the ensemble mean of the median predicted binding affinity from good poses only from Figure 2. A dashed gray line is extended from the mean result of training without imputed labels for easier comparison with the baseline.

The majority of CrossDocked2020, about 60%, is missing a binding affinity label. We theorize that it is potentially not beneficial to have most of the loss affecting the model’s weights come from imputed labels. Thus, we characterized the effect of gradually adding more imputed labels to the training set for the median good only ensemble imputation type. In order to do this, we randomly selected 20% of our imputed labels to be added to the training set, trained five new models with different seeds on a new version of the data set with these extra imputed labels, and measured their performance. We then repeated this with another randomly selected 20%, making 40% total, then again for 60% total, and one last time for 80% total. The results of this experiment are shown in Figure 4 and S4.

We observe a general trend of improvement as more imputed data are added. Notably, at the inclusion of 80% of the imputed binding affinity labels, the improvement gain plateaus. This is interesting as including 80% of the imputed binding affinity labels corresponds to having approximately 47.2% of the poses in the training set with imputed labels and 41.1% of the poses with a known binding affinity label (with the remaining data unlabeled). While not precisely tested here, this result implies that maximal improvement is achieved with a balance of imputed and known labels during training.

### Independent Roche Test Set

We further evaluated our best-performing models on the Roche data set. Notably, since the Roche data set was made available in 2022, none of its ligands are present in CrossDocked2020v1.3. Additionally, none of the exact structures utilized in the Roche data set are present in CrossDocked2020v1.3, but the target, PDE10A, is present in CrossDocked2020v1.3. We evaluated the Roche data sets in two contexts: (1) redocking the provided receptor–ligand pose with Gnina and (2) scoring the provided receptor–ligand pose. The first context provides insights into how well our models perform in a blind drug discovery context, while the second provides insights into how well the models would perform if the “correct” answer is known. Thus, this data set serves as an indicator of how well our recommended imputation procedure would apply in a real-world scenario.

Our best-performing model for test set RMSE was the model trained with the median good only ensemble. During the training procedure of the prior experiments, we trained five models on different random seeds of our three-fold clustered cross-validation splits for each round of imputation, for a total set of 15 models per round of imputation. We then performed two experiments for each context. First, for a given fold of training data, the mean of the five differently seeded models is taken as our prediction for the Roche data set and calculated the RMSE. We can then compare this RMSE with the ones generated by the models that were trained on the same fold but with imputed labels.

The second experiment is set up similarly. Instead of utilizing the ensemble prediction based on the folds of CrossDocked2020v1.3 utilized for training, we instead took the ensemble prediction of all 15 models for the predicted label for the Roche data set and calculated the RMSE. This was performed for the set of models trained without imputed labels, the models trained with one round of the median good only ensemble of imputed labels, and the models trained with two rounds of the median good only ensemble of imputed labels.

The RMSEs of both experiments are listed in Table 3. There is a general trend of imputation improving the model RMSE on the Roche data set. However, the only RMSE improvement of the models trained with imputed data to achieve the statistical significance was those trained on fold 2 of CrossDocked2020v1.3 (*p* < 0.05, as measured by a one-tailed bootstrap of the difference of the means).

**Table 3:** Test Set RMSE on the Roche Data Set Given a Certain Amount of Imputed Training Data^a^

training fold	no imputation	1 round imputation	2 round imputation
(a) Performance on Gnina Docked Poses of the Roche Data Set			
0	1.145	1.128	1.131
1	1.076	1.073	1.077
2	1.260	**1.179**	**1.173**
all	1.134	1.116	1.112
(b) Performance on the Pose Provided by the Roche Data Set			
0	1.131	1.114	1.117
1	1.047	1.048	1.055
2	1.270	**1.169**	***1.155***
all	1.120	1.098	1.093

a For the first three rows, we are reporting the RMSE of taking the mean of the predictions for the five models trained on that fold of CrossDocked2020v1.3 as our prediction for each molecule in the Roche data set. For the final row, we took the mean of all 15 models for our prediction. Numbers in bold are statistically significantly different from the RMSE values reported without imputation. Numbers in italics are statistically significantly different from the RMSE reported with 1 round of imputed training. Significance was determined by bootstrapping the RMSE 10,000 times and testing for overlapping confidence intervals.

## Discussion

We have demonstrated that imputed binding affinity labels improve the model performance at predicting receptor–ligand binding affinity for the CrossDocked2020 data set. Additionally, due to our model’s shared weights for the pose classification and binding affinity regression tasks, we also observe that including imputing binding affinity labels during training has a small beneficial effect on pose classification. We also investigated several imputation approaches utilizing only our CNN model and suggest some best practices on imputation going forward: ensemble-based approaches to ML-generated imputation labels are better (Figure 1 and S1), two rounds of imputation generation achieves maximal improvement (Figures 1, 3, S1 and S3), median good only ensemble is the best approach to generating imputed binding affinity labels (Figure 2 and S2), and a roughly equal number of imputed labels and known labels achieves optimal performance gains (Figure 4 and S4).

Notably, we investigated only utilizing a model trained with the CrossDocked2020 data for our imputation. This is not representative of chemical space and could just be further reinforcing the biases of CrossDocked2020 during the imputation process. A potential solution to this problem would be to utilize a different model, trained on a different, larger data set (e.g., ChEMBL^31^), which could provide more useful imputed binding affinity labels. There are several initial challenges to such an approach: (1) selecting a different training data set without having leakage into CrossDocked2020, (2) selecting an appropriate input representation that works for both the new data set and CrossDocked2020, and (3) selecting a new model architecture for this task. We leave such a study to future work.

Our first set of experiments shows that imputing a binding affinity label for every pose (individual and individual ensemble) has a small but beneficial effect on the binding affinity prediction task (Figure 1). We then demonstrate that selecting a single imputation label for the entire pocket–ligand pair performs better than our individual ensemble for the binding affinity prediction task’s Pearson correlation (Figure 2b). However, for the binding affinity prediction task RMSE, the median good only ensemble imputation type outperformed the individual and individual ensemble methods (Figure 2a). Additionally, we investigated only some simple approaches to selecting our imputed binding affinity label (minimum, maximum, and median) when selecting a single label for a pocket–ligand complex. It is entirely possible that a more sophisticated approach, such as something related to SICE^22^ or maximum likelihood estimation, could provide better imputation labels. However, these approaches would require a model that also outputs its confidence in its predictions, which is outside the scope of our current Default2018 architecture. Again, we leave such a study to future work.

Our final set of experiments show that utilizing our median good only ensemble imputation type for training also results in small improvements of RMSE on the external Roche data set (final row of Table 3a,b). However, the differences in RMSE were only statistically significant for models trained with fold 2 of CrossDocked2020v1.3. Reassuringly, we see the same trend of performance upon utilizing our networks to evaluate docked poses from Gnina, as simply scoring the pose provided by the data set. Even though the performance trend is similar in both of our experimental contexts for the Roche data set, the general lack of statistical significance suggests that the performance gain of training on imputed labels for a particular data set does not necessarily carry over to new unseen data sets. Again, it is possible that a more sophisticated imputation approach would improve the performance of models trained with imputed labels and could help with this problem. We leave such a study to future work.

Additionally, we observed that imputing a unique binding affinity for each pose (individual and individual ensemble) resulted in a small but statistically significant improvement on the pose classification task (Figure S2 and Table S1). This is not necessarily surprising due to our model’s training procedure. During training, we backpropagate the losses through the output layers and into the CNN. By including imputed binding affinities that are different for every pose, we allow the binding affinity hinge loss to affect all of the poses in the training set. Previously, this loss was only applied to complexes with a known binding affinity. With the different binding affinity labels for each of the poses, we are also including some extra binding affinity information to affect the CNN, which generates the input for both output layers. This can allow the CNN to gain some additional information to help in its pose-classification task.

We also note that our performance gains, while statistically significant for most, but not all, metrics (Tables 2 and S1), were small (e.g., on the order of an improvement of 0.01 on the binding affinity RMSE and Pearson *R*), and so a reasonable perspective is that it is not worth doing. However, unlike approaches that increases model complexity in order to achieve similar improvements in the performance,^3,9−13^ imputing missing labels is an orthogonal technique to model architecture optimization and imposes no increase in model complexity or run-time performance. As long as including imputed labels does not worsen the predictive performance of the model, there is no downside to the end user. Furthermore, if imputed labels are already available or if they can be generated dynamically during training (as opposed to the iterative processes described here), there is little overhead to including them at training time.

We have demonstrated that imputing labels for receptor–ligand binding affinity prediction can improve CNN-based ML models for this regression task. Our best-performing imputation technique, the median good only ensemble, achieved a small but statistically significant improvement on the RMSE and Pearson’s *R* between the true binding affinities and the predicted binding affinities of CrossDocked2020v1.3 (Figure 2 and Table 2). We also provide initial best practices that may apply to future work: utilizing an ensemble of predictions over only the poses that are known to be good, utilizing a roughly balanced amount of known labels with imputed labels for training, and performing our training-imputation cycle twice for the maximal model performance. Though training on imputed labels only provided a small improvement to binding affinity regression, it shows promise as a way to improve ML model training with negligible effect on training time and compute resources, and more studies to explore better imputation approaches and incorporating outside data sources are needed. The code, data splits, and imputation labels utilized in this paper are freely available at https://github.com/francoep/ImputationPaper.

## Supporting Information Available

Student’s *t*-test *p*-values for the coefficient of determination; AUC and Top1 analysis of the difference between 0 and 1 round of imputation for each imputation type; and same experimental setup, analyzing the binding affinity coefficient of determination (*R*^2^) and the binding pose AUC and Top1 (PDF)

## Notes

All data, commands, and notebooks utilized in the creation of this manuscript are available at https://github.com/francoep/ImputationPaper. Source code, training scripts, and model definitions are available at the GNINA project homepage https://github.com/gnina under a dual GPL and Apache license.
