# Supplementary material for: Expanding Training Data for Structure-Based Receptor–Ligand Binding Affinity Regression through Imputation of Missing Labels
Source: ACS Omega. 2023 Oct 26;8(44):41680–8. doi: 10.1021/acsomega.3c05931 (PMC10634251; doi:10.1021/acsomega.3c05931)
Supplement: Supplementary file 1 — ao3c05931_si_001.pdf [file ao3c05931_si_001.pdf]

Supporting Information: Expanding Training Data for Structure-based Receptor-Ligand Binding Affinity Regression Through Imputation of Missing Labels

Paul G. Francoeur* and David R. Koes

Department of Computational and Systems Biology, University of Pittsburgh, Pittsburgh, PA 15260

E-mail: paf46@pitt.edu

Table S1: Student's T-test p-values for the difference between 0 and 1 round of imputation for each of the methods. This table corresponds to the data utilized to generate Figure S2. Notably, for the binding affinity RMSE, every method results in a statistically significant difference as compared to not performing the imputation. This is not true for all the other metrics, though generally imputation results in a statistically significant difference for binding affinity Pearson's R and AUC while generally failing to produce a statistically significant difference for Top1.

Imputation Type	R^2	AUC	Top1
Individual	0.442	0.000747	0.459
Individual Ensemble	0.106	0.000755	0.0433
Median Ensemble	0.0152	0.00477	0.391
Median Good Only Ensemble	0.331	0.0131	0.485
Max Ensemble	0.00574	0.352	0.914
Max Good Only Ensemble	0.165	0.00429	0.130
Min Ensemble	3.13e-06	0.00488	0.00358
Min Good Only Ensemble	0.0311	0.0218	0.324

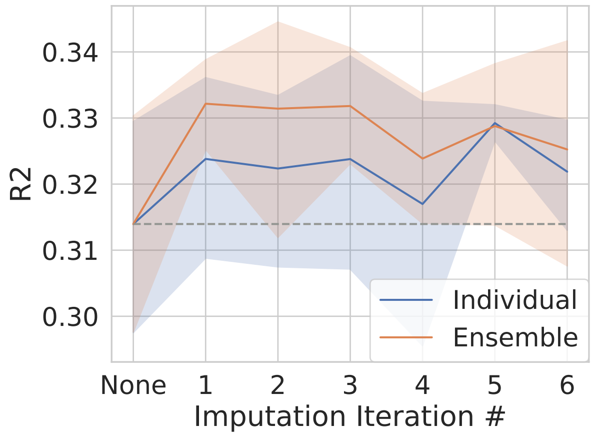

(a) Binding affinity coefficient of determination improves with the addition of imputed labels. Higher is better.

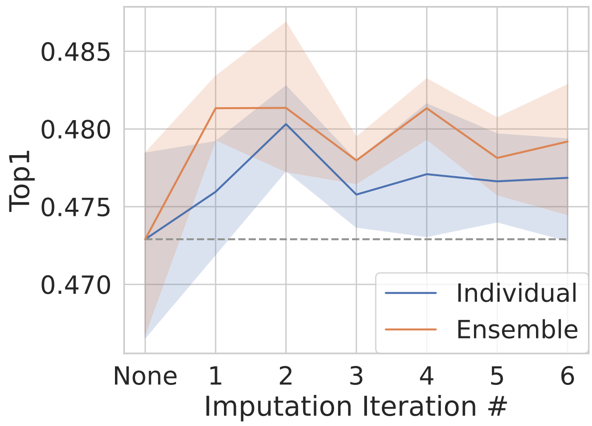

(b) Binding pose Top1 improves with the addition of imputed labels. Higher is better.

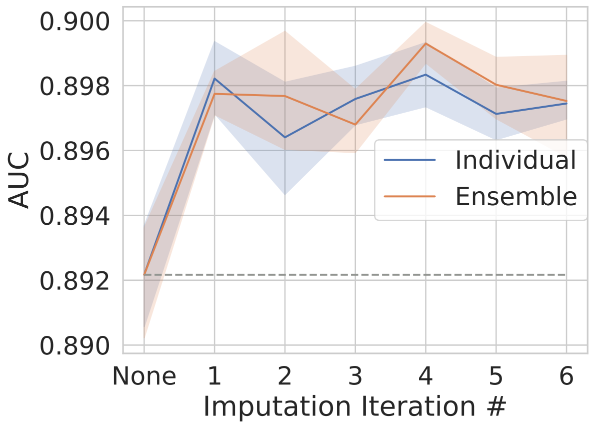

(c) Binding pose AUC improves with the addition of imputed labels. Higher is better.

Figure S1: Binding affinity coefficient of determination and binding pose classification results. Training on imputed data has a small improvement on all metrics. At each data point we plot the mean of 5 models trained with different random seeds. The colored area is the 95% confidence interval around the mean calculated via bootstrapping in *seaborn*. The blue line shows the results of five different random seeds (Individual Table 1). The orange line shows an ensemble approach, taking the mean of the five models as the imputed label of every pose (Individual Ensemble Table 1). A dashed grey line is extended from the mean result of training without imputed labels for easier comparisons.

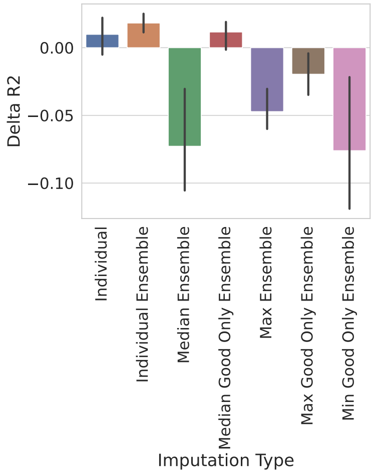

(a) Change in binding affinity coefficient of determination relative to no imputation with a variety of imputation styles. Higher is better.

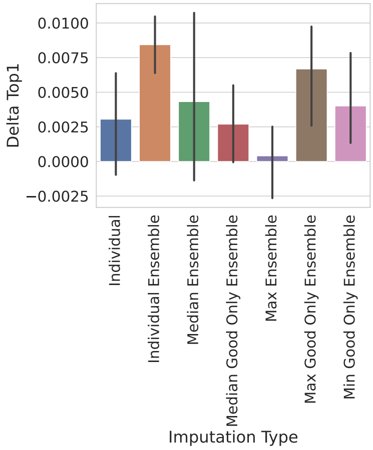

(b) Change in pose classification Top1 relative to no imputation with a variety of imputation styles. Higher is better.

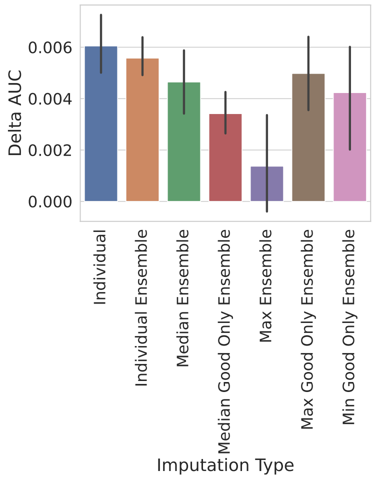

(c) Change in pose classification AUC relative to no imputation with a variety of imputation styles. Higher is better.

Figure S2: Comparing different binding affinity imputation styles. The performance metrics for five models with different seeds trained with each imputation style were subtracted from the mean performance of training without imputation. The shaded area is the 95% confidence interval of this described value calculated via bootstrapping in *seaborn*. For each plot, a bar corresponds to a singular imputation style. The first two styles, Individual and Individual_Ensemble are the same that are used in Figure S2. For the rest of the styles, we select one number for each pocket-ligand pair, either by the median (Med), maximum (Max), or minimum (Min). We then either utilized the Med/Max/Min of all poses, or only the poses labeled good. The Min Ensemble results were omitted, due to performing so poorly that they re-scaled the plots (Delta R^2 -2.59 Delta Top1 -0.0175, and Delta AUC 0.00219). The Student's T test for each of these values is reported in Table S1. A dashed grey line is extended from the mean result of training without imputed labels for easier comparisons.

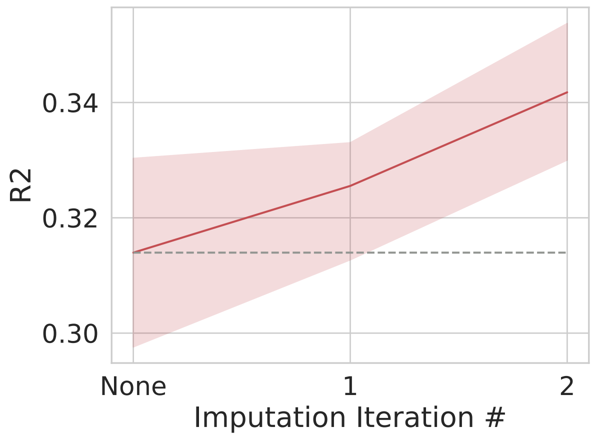

(a) Binding affinity coefficient of determination improves when utilizing an ensemble of the median predicted affinity for imputation. Higher is better.

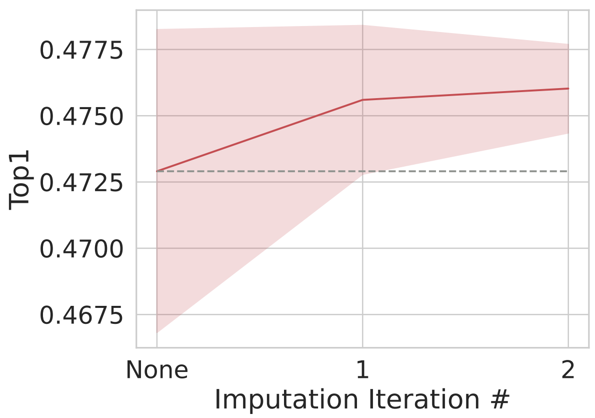

(b) Binding pose Top1 improves when utilizing an ensemble of the median predicted affinity for imputation. Higher is better.

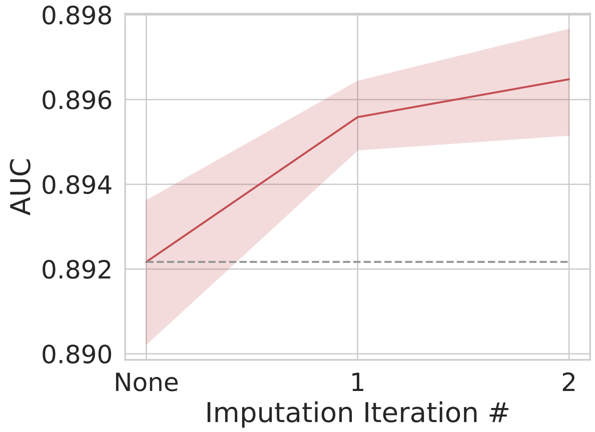

(c) Binding pose AUC improves when utilizing an ensemble of the median predicted affinity for imputation. Higher is better.

Figure S3: Performance metrics of our best imputation approach, taking the ensemble mean of the median predicted binding affinity for each pocket-ligand complex good pose, for binding affinity regression. The 0th point on the line is the model results after training on the original dataset. We then used that model to generate the imputed labels, and utilized them to train the model for the 1st data point. Said model was then used to generate the imputed labels for the second datapoint's model's training. For each point five models with different seeds were trained from scratch. The shaded area is the 95% confidence interval of the mean calculated via bootstrapping in *seaborn*. A dashed grey line is extended from the mean result of training without imputed labels for easier comparisons.

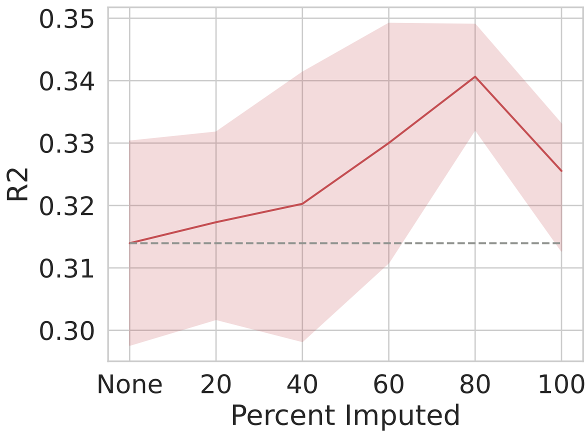

(a) Binding affinity coefficient of determination improves as imputed data is added to the training set. Higher is better.

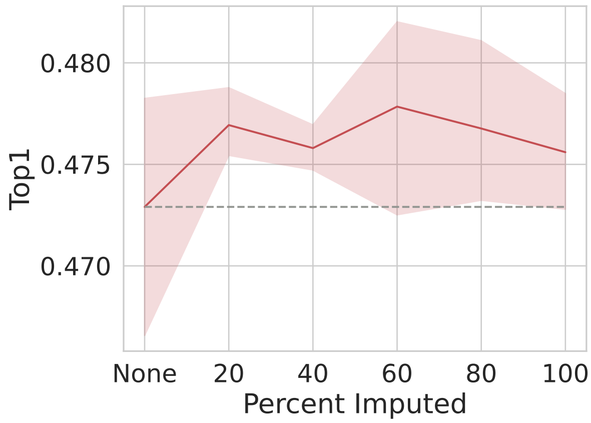

(b) Binding pose Top1 exhibits a small improvement with imputed data during training. Higher is better.

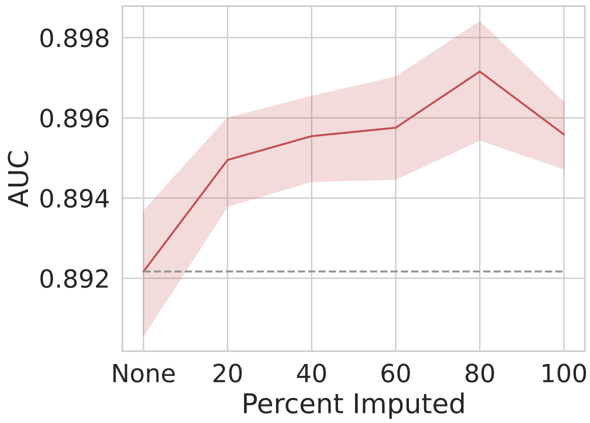

(c) Binding pose AUC improves as imputed data is added to the training set. Higher is better.

Figure S4: Effect on metrics as a function of successively adding more imputed binding affinities to the training set. Each plot is showing the results of five models with different seeds, being trained on successively more of the imputed binding affinity labels. The shaded area is the 95% confidence interval of the mean calculated via bootstrapping in *seaborn*. Shown are no imputed labels, to all of the imputed labels, in increments of 20%. The imputation generation procedure is the ensemble mean of the median predicted binding affinity from good poses only from Figure S2. A dashed grey line is extended from the mean result of training without imputed labels for easier comparisons.
